# The Gardner Case—Correction

**Published:** 1879-12

**Authors:** 


### Editorial.

### THE GARDNER CASE—CORRECTION.

In some editorial remark in the November number of the Register, in reference to the alleged arsenic poisoning of Mr. Gardner in dental treatment, some surprise and regret was expressed at the statement of opinions of physicians and dentists, as given in the various New York papers. That these reports were garbled or exaggerated would hardly fail at first sight to convince any reasonable mind. In verification of this statement, the following from Prof. Abbott is given; taken from the proceedings of the Second District Dental Society of New York, as published in the *Dental Miscellany*.

Dr. Abbott says: "I feel very much hurt over this article which has appeared in the *Times*, and would like to explain it. At half-past four o'clock on Monday, 6th October, when I arrived at the College to my lecture, the reporter was there, and commenced talking upon the subject of the Gardner case in Brooklyn, and asked my opinion in reference to it. I said I had no opinion to give, as I knew nothing about the case at all. I further told him that I had no time to talk with him upon the subject as my lecture hour had arrived, and I would be occupied an hour. He then asked if I would have any time after my lecture. I said, No; as I must hurry home to my dinner at six o'clock. Then he says, "I will come and walk or ride up with you, and talk the matter over." I said, "Very well, if you choose to do that, I will talk with you on the way up. But I have nothing to say in reference to this case, because I know nothing about it. I would not express an opinion in reference to it. I know none of these gentlemen in Brooklyn who are said to have had the case in charge, and who have pronounced it a case of arsenical posioning. "Well," he said, "It is not that case particularly I want to know about, but it is the use of arsenic." "Very good, I can give you what is done with arsenic as a general thing." I then left him and went to my lecture. After the lecture, I met him on the street, and we walked up to the corner of 40th street and Madison Ave., together. The conversation all the way was upon this matter of arsenic and its effects. And he was very persistent in his question, all the way up, in reference to the action of arsenic; wanting to know if it would do this, and do that; if it would effect such a thing, and effect such a thing. Now, if I may be allowed, I would like to read in this article what I am reported to have said and follow it up with what I really did say. I am reported as saying. "Those, who too easily deny that Mr. Gardner died from the effects of arsenic placed in his tooth, make this mistake: they fail to distinguish between arsenical poisoning through the stomach and arsenical poisoning by direct contact with the blood."

"The question put to me was: 'How does arsenic affect the system when placed in the mouth, as compared with its effects when taken into the stomach?' I stated to him that the effects in the mouth were altogether local, unless the quantity placed there was very large; in that case absorption might take place so that we would get some of the symptoms we usually find when taken into the stomach. But the effect of arsenic, as a general thing, was directly upon the surface with which it came in contact, penetrating under the surface just so far as the quantity and strength of the material would permit. Then, if absorption took place, as it did sometimes, we would get some of the symptoms of arsenical poisoniug through the stomach, such as extreme nausea, retching, inability to retain anything on the stomach—occasional diarrhœa—in fact, nearly all the symptoms, with the exception of acute gastritis Those we might get from arsenic as placed locally. Then I am made to say, 'It is the universal practice with dentists to place arsenic in the teeth to kill nerves.' I said they did not do that—all of them. There are a great many dentists who do not place arsenic in the teeth, and think it is a great sin and shame that it should be done. In fact, I don't place arsenic in teeth myself—have not done so twice in ten years 'It is taught in all dental colleges,' he makes me say. The subject of dental teaching was not spoken of at all in that interview—not a word said about it one way or the other. Speaking of the manner of introducing arsenic into teeth for the purpose of destroying pulps, he makes me say, 'Any operator is liable to dip to deeply, and even to lift more than enough of the poison to kill the strongest man, if it should get to his blood.' The amount of tissue destroyed would depend entirely upon the amount of arsenic placed in contact with, and held upon the gum. Nothing whatever was said about destroying life, from the application of arsenic to a tooth to destroy the pulp, in this connection. What I did say was this: 'That if the arsenic was carelessly put into a tooth and came out, and came in contact with the periosteum surrounding the root of the tooth, it would destroy the gum, periosteum and bone as far as the strength of the material would admit—whether it be a small particle or a large amount, it would destroy in proportion to the amount of poison there was there. He makes me say in another place, 'I have known a great many instances of poisoning from treatment of the teeth, but in all of them proper treatment in time saved the patient, and in most of them there was not enough arsenic to be fatal.' What I did say, was this: 'I have seen a great many cases of arsenical poisoning from the treatment of the teeth, but in none of them was there arsenic enough to produce anything but local disturbances.

"Then, again, he makes me say: 'In all of these cases, so far as they went, the symptoms were about the same as the symptoms in the Gardner case." Now, what I said in that connection was, that if the statements in the paper be correct, that the man's mouth and face were black, and that the parts were sloughing off, then the symptoms were those of arsenical poisoning. 'In another place he makes me say this: I would say this, all the symptoms in the Gardner's case, but two, go to show death by arsenical poisoning."

"Now, what I did say, was this: From what I learn in conversation with you, I should judge that Mr. Gardner had died from the effects of an alveolar abscess." Then he says: "What symptoms are there of alveolar abscess?" Said I: "You have just told me that the man's face—or as you call it, gland—was swollen way down upon his neck—you might say upon his shoulder; that is one symptom of an alveolar abscess. You have just told me again that his jaw was very rigid and could not be moved one way or another; that is another symptom of alveolar abscess. You have said, or it was published, that Dr. Lewis opened a little pustule in the man's mouth which discharged a very putrid fluid. These symptoms are all of them prominent symptoms pointing to alveolar abscess."

"Then, he says: 'Could this abscess not have been accelerated by the arsenic?' I said, 'No; arsenic could not have anything to do whatever with the origin or developmeut or acceleration, if you choose, of an alveolar abscess.' "

"Now, I want to make one statement more to you before I leave you, and that is, I am strongly impressed with the idea that that man died from an alveolar abscess improperly treated; and my opinion is, if he had kept Dr. Marvin, done as he had told him, and allowed him to treat his tooth as he wanted to, he would have been a live man to-day."

It is very gratifying to present this correction of the alleged statement of Dr. Abbott. Occupying the position of a teacher, he could hardly afford, on his own account, as well as that of the institution which he represents, to be misrepresented, as he was in the reports of his remarks upon this case.

It is too often the case that when newspaper reporters desire to produce a sensation, they proceed without regard to truth, decency, or the reputation of other people. This is a nuisance about which it is of no avail to scold—it must be endured.

